# Ontogenesis of oxytocin pathways in the mammalian brain: late maturation and psychosocial disorders

**DOI:** 10.3389/fnana.2014.00164

**Published:** 2015-01-20

**Authors:** Valery Grinevich, Michel G. Desarménien, Bice Chini, Maithé Tauber, Françoise Muscatelli

**Affiliations:** ^1^Schaller Research Group on Neuropeptides, German Cancer Research Center and CellNetwork Cluster of Excellence of the University of HeidelbergHeidelberg, Germany; ^2^Institute of Functional Genomics, Centre National de la Recherche Scientifique, Institut National de la Santé et de la Recherche Médicale, Université Montpellier 1, Université Montpellier 2Montpellier, France; ^3^Consiglio Nazionale delle Ricerche Institute of NeuroscienceMilan, Italy; ^4^Reference Centre for Prader-Willi Syndrome – Department of Pediatric Endocrinology, Hôpital des Enfants Centre Hospitalier Universitaire de Toulouse 330Toulouse, France; ^5^Institut National de la Santé et de la Recherche Médicale Unité Mixe de Recherche 1043, Paul Sabatier University Toulouse IIIToulouse, France; ^6^Institut de Neurobiologie de la Méditerranée Unité Mixe de Recherche U901, Institut National de la Santé et de la Recherche Médicale, Parc Scientifique de LuminyMarseille, France; ^7^Aix-Marseille Université, Institut de Neurobiologie de la Méditerranée Unité Mixe de Recherche 901Marseille, France

**Keywords:** oxytocin, oxytocin receptor, ontogenesis, somatodendritic release, axonal release, autisn, Prader-Willi syndrome

## Abstract

Oxytocin (OT), the main neuropeptide of sociality, is expressed in neurons exclusively localized in the hypothalamus. During the last decade, a plethora of neuroendocrine, metabolic, autonomic and behavioral effects of OT has been reported. In the urgency to find treatments to syndromes as invalidating as autism, many clinical trials have been launched in which OT is administered to patients, including adolescents and children. However, the impact of OT on the developing brain and in particular on the embryonic and early postnatal maturation of OT neurons, has been only poorly investigated. In the present review we summarize available (although limited) literature on general features of ontogenetic transformation of the OT system, including determination, migration and differentiation of OT neurons. Next, we discuss trajectories of OT receptors (OTR) in the perinatal period. Furthermore, we provide evidence that early alterations, from birth, in the central OT system lead to severe neurodevelopmental diseases such as feeding deficit in infancy and severe defects in social behavior in adulthood, as described in Prader-Willi syndrome (PWS). Our review intends to propose a hypothesis about developmental dynamics of central OT pathways, which are essential for survival right after birth and for the acquisition of social skills later on. A better understanding of the embryonic and early postnatal maturation of the OT system may lead to better OT-based treatments in PWS or autism.

## Introduction

Oxytocin (OT) is a neuropeptide which is synthesized in defined nuclei of the hypothalamus, paraventricular (PVN), supraoptic (SON), and accessory (AN) nuclei and secreted both in the blood circulation as a hormone and in the brain as a neuromodulator. During the last decade, hundreds of original reports and reviews have been published, clearly showing that the OT system is a key regulator of all the aspects of social behavior, including those involved in reproduction and care of the offspring (Lee et al., 2009). Notably, in humans, OT facilitates the processing of social information and improves cognitive emphatic abilities, representing a potential new approach for the treatment of autism spectrum disorders, as extensively reviewed elsewhere (Meyer-Lindenberg et al., 2011). Even if OT is currently regarded as a “prosocial” drug, this does not implicate *per se* a “positive” connotation. OT has been found to increase trust and generosity (Kosfeld et al., 2005), but also gloating and envy (Shamay-Tsoory et al., 2009), which are also manifestations of complex “social” behaviors. Similarly, OT increases in-group but not out-group cooperation, an ethnocentric behavior that can promote prejudice, xenophobia, and intergroup violence (De Dreu et al., 2011). Furthermore, OT has been found to induce adverse behavior in borderline-personality disorders (Bartz et al., 2011). These results suggest that the selection of patients will play a crucial role in determining the outcome of the symptomatic treatment with this neuropeptide, the use of which should be restricted to those individuals who could benefit from it. An understanding of the OT-mediated cellular and molecular mechanisms on neuronal networks underlying social and feeding behavior is needed for a successful employment of this peptide in neuropsychiatric and genetic disorders. However, despite the large body of knowledge about physiology and central pathways of OT (see reviews from Landgraf and Neumann, 2004; Ross and Young, 2009; Lee et al., 2009; Knobloch and Grinevich, 2014), the developmental aspect of the OT system remains the *“locus minoris”* of neuroscience and neuroendocrinology. Here, we collected virtually all of the available information, including our personal ongoing research, and provide some general conclusions regarding the dynamics of central OT pathways in prenatal and early postnatal ontogenesis. Furthermore, we clearly demonstrate, by investigating a mouse model for the Prader-Willi syndrome (PWS), that changes in the developmental maturation of the OT system cause metabolic and social alterations. These can be significantly improved or even fully compensated by OT treatment in the first days after birth, opening the door for a powerful pharmacological therapy for PWS in early infancy.

## The birth of OT-ergic nuclei

**Figure 1:**
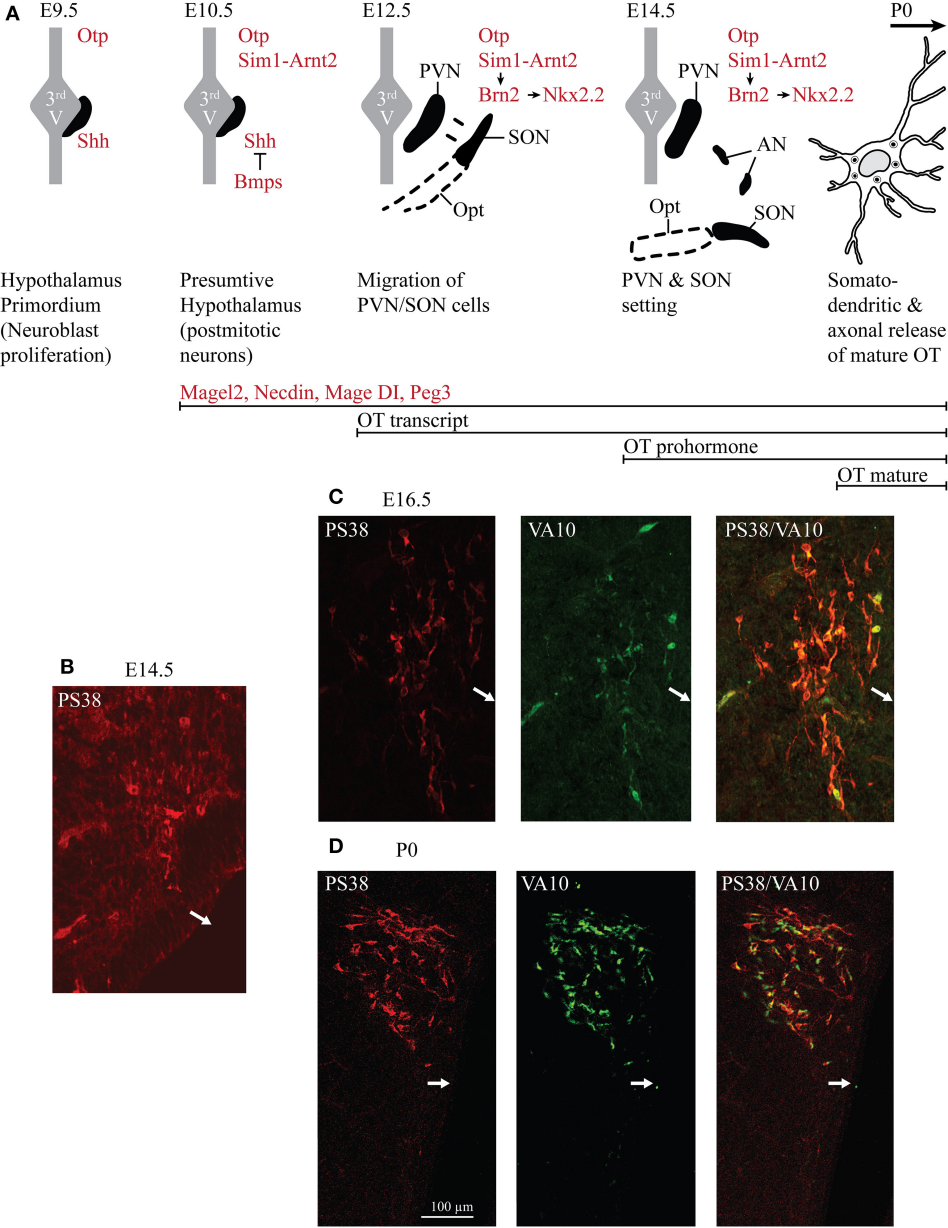
**Ontogenesis of the OT neurons during mouse embryonic development**. **(A)** The developmental stages and transcription factors (in red in the drawings) known to be involved in the setting of the PVN, SON, and AN. Below are indicated several genes known to be expressed in OT neurons and the inactivation of each of these genes alters OT neurons. The developmental stages from which an expression of *OT* transcript, OT prohormone and OT-mature form are detected are also indicated (in black). **(B)** Immunohistochemical detection of the OT-neurophysin I (associated with OT prohormone) using the PS38 antibody (in red) and of the OT-intermediate forms using the VA10 antibody (in green). At E12.5 pcd, we did not detect any signal (not shown). At E14.5 pcd we first detect few cells expressing the OT-neurophysin I **(B)**, probably the OT-prohormone, but not OT intermediate forms. **(C)** At E16.5 pcd we detect both OT-neurophysin I and the OT intermediate forms. **(D)** At the day of birth virtually all OT neurons of the PVN co-express both OT-neurophysin I and immature forms of OT. White arrows indicate the location of the third ventricle.

In many species (rodent, human, zebrafish, chicken), all oxytocin (OT) neurons are generated from the proliferative (“convoluted”) neuroepithelium of the diamond shaped third ventricle (Table 1 and Figure 1). Birth-dating studies revealed that these hypothalamic neurons are generated in the second half of the gestational period in rodents, within the first quarter of the gestational period (E30-43; length of pregnancy ~165 days) in macaques (Markakis, 2002) and at the middle of pregnancy in humans (Swaab, 1995).

**Table 1:** **Birth of OT neurons and OT synthesis**.

**Biosynthesis**	**Mouse**	**Rat**	**Primates/Human**
Birth of OT neurons	E10.5-12.5: postmitotic neurons giving rise to the PVN, SON, AN (Caqueret et al., 2006)	E13-E15 for the PVN	In macaques:
	E13.5-E15.5: terminal differentiation of PVN and SON neurons (Caqueret et al., 2006)	E13-E14 for the SON (Altman and Bayer, 1978a,b,c)	E30-E38 for the PVN
			E40-E43 for the SON (van Eerdenburg and Rakic, 1994)
Initial OT gene expression (based on *in situ* hybridization)	E15.5	E16-E17 (SON, PVN) (Laurent et al., 1989; Trembleau et al., 1995)	15 Weeks of human gestation (Allan Brain Atlas)
	E15.5 (presumptive PVN) and E18.5 (SON) (Allan Brain Atlas, Jing et al., 1998)		
Initial synthesis of pre-proOT (based on immunohistochemistry)	E14.5	E16 (PVN, SON) (Whitnall et al., 1985; Altstein and Gainer, 1988)	ND
	PVN, SON, AN (Figure 1 of this review)		
Initial production of mature form of OT	P0 (birth) in PVN, SON, AN (Schaller et al., 2010)	E21-P0 (SON, PVN, AN) (Boer et al., 1980; Buijs et al., 1980; Sinding et al., 1980; Whitnall et al., 1985; Altstein and Gainer, 1988)	14 Weeks of gestation (Schubert et al., 1981; Goudsmit et al., 1992; Swaab et al., 1995)

In rodents, the SON and PVN appear very early. At embryonic day (E) 12.5 dpc (days post-coîtum), two groups of cells are identified in the mouse: one near the third ventricle and the other moved lateral to the surface pial to give rise to the SON (Dongen and Nieuwenhuys, 1989). At E14.5 dpc, the PVN and the SON are settled (Nakai et al., 1995), while AN are recognized later (probably due to their small size and relatively small number of cells; Altman and Bayer, 1978a,b,c). At this stage, an antibody recognizing the Neurophysin-I (the carrier protein for OT) reveals a positive immunosignal (Figure 1B), consistent with the expression of the OT-prohormone, not yet detected at E12.5 dpc. Furthermore, an antibody recognizing the intermediate forms of OT (VA10) reveals a positive immunosignal of the OT-intermediate forms at E16.5 dpc (Figure 1C), but not at E14.5 dpc. Importantly, the mature, amidated form of OT is detected at birth (see Table 1) and co-exists with immature forms of OT during the entire postnatal life (Figure 1D). The role of an earlier production of the non-mature forms of OT has not been studied, although a functional role of these forms during the embryonic development has been suggested (Tribollet et al., 1988).

In humans, the SON and PVN are completely formed at 25 weeks of gestation (Dörner and Staudt, 1972) and OT-immunoreactivity is first detected at the age of 26 weeks (Wierda et al., 1991). At that age, the number of stained OT neurons is relatively similar in the fetal and adult hypothalamus (Van der Woude et al., 1995), while the morphological analysis of individual magnocellular neurons suggests that these cells are still immature, as can be seen by the gradual increase of their nuclear volume (Rinne et al., 1962; was also observed in the rat by Crespo et al., 1988).

## Genetic hierarchy of OT neuron formation

The signaling molecules and transcription factors that are involved in the determination and differentiation of OT neurons are not well known, only a few mouse studies reported factors involved in the early stages of development of the hypothalamic-neurohypophyseal system (Carrel and Allen, 2000; Caqueret et al., 2006; Szarek et al., 2010).

The early patterning of hypothalamus has been shown to depend on a cascade of transcription factors (Figure 1A): firstly the sonic hedgehog (SHH) is necessary to establish the hypothalamus primordium (E9.5 dpc in mouse) (Mathieu et al., 2002; see also Blaess et al. (2014) in this Research Topic of Frontiers in Neuroanatomy); the local production of the bone morphogenetic proteins (BMPs) is then required to down-regulate SHH and establish region specific transcription profiles (Ohyama et al., 2008). The bHLH-PAS (basic helix-loop-helix PER-ART-SIM) transcription factor SIM1 is expressed in the incipient PVN, SON and AN from E10.5 dpc (Caqueret et al., 2006) where it dimerises with ARNT2 (Michaud et al., 2000; Hosoya et al., 2001). A key downstream target of SIM1/ARNT2 is Brn2, a POU domain transcription factor required for OT (as well as for two other types of neuroendocrine neurons, expressing arginine-vasopressin (AVP) and corticotropin releasing hormone neurons of the PVN/SON/AN and expressed from E11.5 dpc, Nakai et al., 1995; Schoneman et al., 1995). In a parallel or convergent SIM1/ARNT2 pathway, the homeobox orthopedia (OTP) factor is also necessary for Brn2 expression (Caqueret et al., 2006), which is still expressed at E15.5 dpc, with Nkx2.2. All of these factors are required to define at E12.5 dpc the prospective PVN domain, however, from this stage on, the factors that will specify the parvocellular^1^ and magnocellular OT neurons have not been identified. The ablation of Brn2 results in a loss of all neurons of the PVN, SON and presumably of the AN (Nakai et al., 1995; Schoneman et al., 1995). Importantly the lack of axonal projections of magnocellular OT and AVP neurons in the Brn2 knock-out mice to the pituitary (Schoneman et al., 1995), as also observed in the Arnt2-knockout mice (Hosoya et al., 2001), leads to progressive loss of pituicytes (pituitary astrocyte-like glial cells). All together, these results suggest a role of OT and/or AVP in the formation of the neurohypophysis. Indeed, in the absence of OT, the neurovascular interface in the neurohypophysis does not form in zebra fish (Gutnick et al., 2011).

One approach to identify such factors involved in the determination and differentiation of OT cells is the analysis of the OT promoter and its upstream and downstream sequences. Interestingly, genomic DNA constructs, including more than 500 base pairs (bp) upstream and 3.6 kb downstream of the OT gene, enabled an expression of OT or EGFP-reporter in OT-neurons only (Young et al., 1990; Belenky et al., 1992) in adult transgenic rodents. Later, a genomic analysis of the OT-promoter (500 bp) defined a minimal promoter sufficient for expression in OT-neurons (magnocellular neurons) of adult rodents (Fields et al., 2012; Gainer, 2012). Several binding sites for transcription factors (ERE, COUP-TF, SF1) have been identified in this sequence, but not the ones for the transcription factors described above (Figure 1A), suggesting that the DNA sequence required for the development of OT cells is not present in these 500 bp.

In conclusion, from the E12.5 dpc stage, the transcriptional factors that will specify the parvocellular and magnocellular OT neurons have not yet been characterized.

Several other genes such as *fibroblast growth factor 8* (Brooks et al., 2010), the *Mage-D1*, *Necdin* and *Magel2* genes, *CD38*, *Peg3* (Figure 1 and **Table 3**), are known to be expressed in mouse hypothalamus during development. Their knock-out (KO) alters the number and/or function of OT-neurons, but their role in OT-neurons is not clarified yet.

## The biosynthesis of OT in ontogenesis

**Figure 2:**
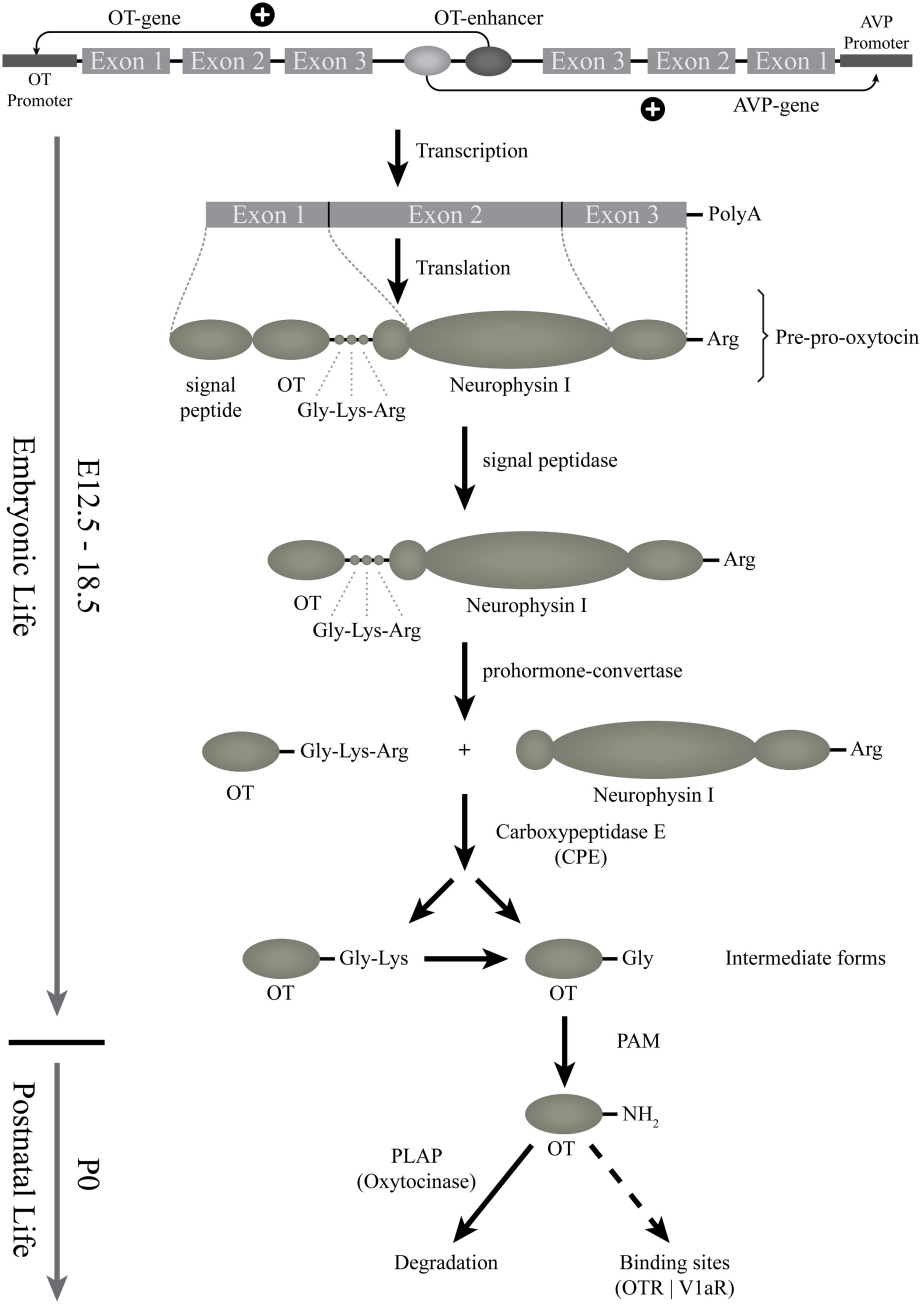
**Starting from the gene: the biosynthesis of the different forms of OT**. This scheme represents the genomic structure of the OT gene, which is contiguous to the AVP-gene; regulatory elements might be shared. The transcription and translation steps of the *OT* gene are then indicated. First, OT preprohormone is produced, that will be cleaved and matured by successive enzymes. The OT intermediate forms are produced from E16.5 (see above) but the mature amidated OT form is detected only from birth. The released mature form is then degraded by an oxytocinase (PLAP) or binds to OT binding sites (OTR or V1a-receptors). PAM, alpha-amidating monooxygenase; PLAP, placental leucine aminopeptidase.

In the rat and mouse, the OT can be detected by several techniques (such as radioimmunoassay, enzyme immunoassay, immunohistochemistry) from the beginning of the second gestational week (Table 1). However, all of these techniques are based on the use of OT antibodies. The specificity of these antibodies, in particular against the different forms of OT, is not always established. The most reliable and relevant studies have been performed with the use of antibodies characterized by Harold Gainer's laboratory or using Mass Spectrometry Ànalysis, which, unfortunately, has not been used in developmental studies. Based on the tools used for OT detection, the OT prohormone is found in embryos just after the appearance of the OT mRNA, while the mature OT peptide is only released from birth on (Table 1 and Figure 1). The OT gene encodes for the Pre-Pro-OT-Neurophysin I (pre-pro-hormone), which is cleaved by different enzymes to give rise to different OT intermediate forms and to the Neurophysin I, and finally to the mature amidated form that is released (Figure 2). It has been shown that the steady state of the mature OT form can be controlled by an oxytocinase (P-LAP) that is produced in periphery and centrally by the OT-magnocellular neurons. Noticeably, P-LAP is also expressed in parvocellular OT neurons and in other brain structures (Tobin et al., 2014).

Before birth, there is a delay in OT prohormone processing with an accumulation of the OT intermediate forms in OT neurons until birth. This delay was not observed for AVP processing as the amidated AVP form is detected as early as E16.5 dpc (Whitnall et al., 1985; Altstein and Gainer, 1988). Furthermore, morphological and electrophysiological properties of the magnocellular OT neurons are not mature at birth; their morphological and electrophysiological properties develop progressively during the first two postnatal weeks (see below, as well as Swaab, 1995, 1997a,b). Thus, although functional at birth, the OT-ergic system undergoes a progressive maturation during early postnatal life and is not mature before weaning in rodents and, most likely, in human infants as well. However, a comparison of immature and mature forms of OT in human development has never been studied.

## Electrical properties of OT neurons after birth

**Figure 3:**
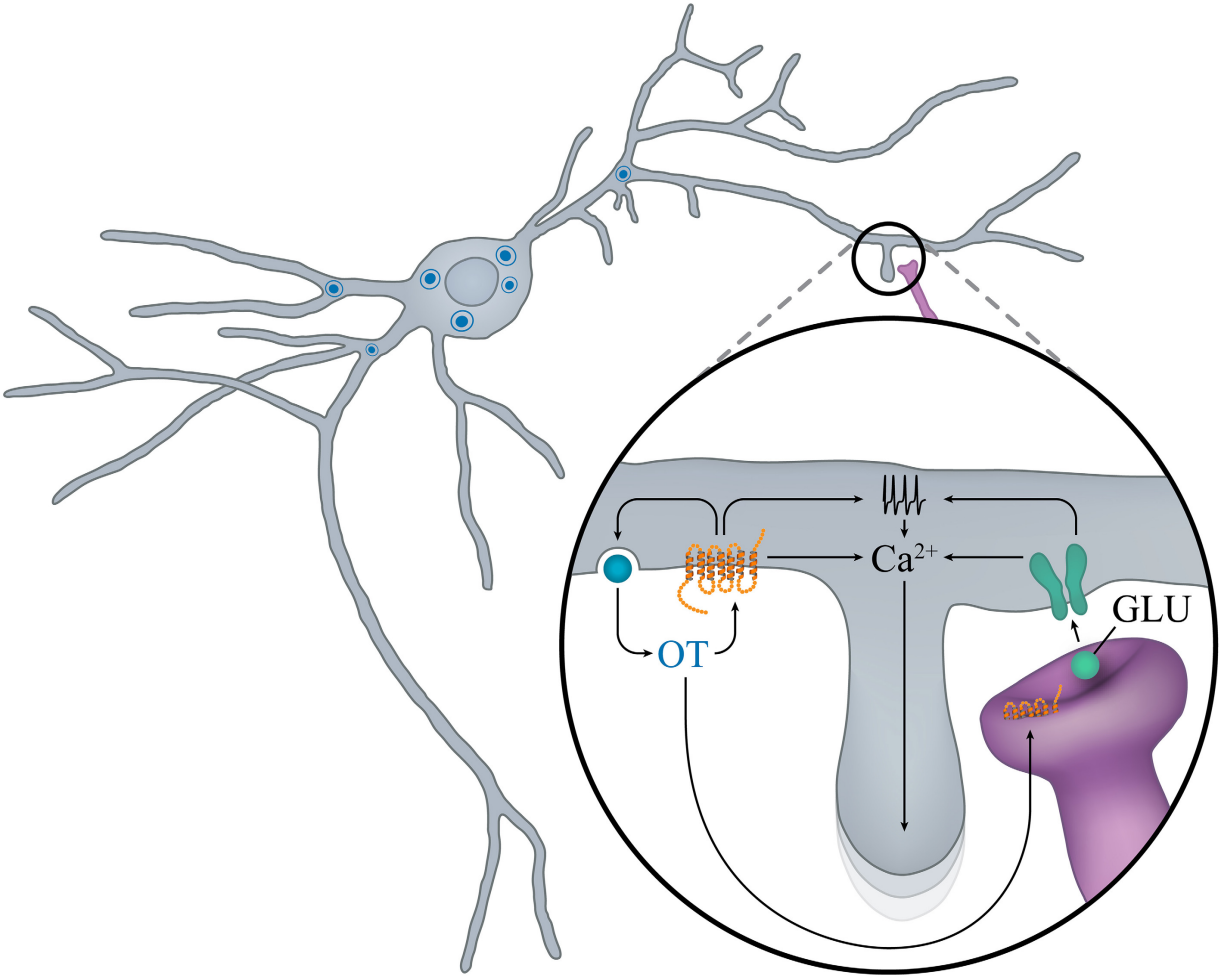
**“Auto-control” of OT neurons in early postnatal ontogenesis**. In the rat SON during the second PN week, locally released OT promotes calcium mobilization and OT release, and favors the maturation of glutamatergic inputs. Activation of NMDA and OTR increases electrical activity and mobilization of calcium from intracellular stores and promotes growth of new dendritic branches.

To our knowledge, no electrophysiological studies have been performed on embryonic OT neurons. However, the groups of Francoise Moos and Michel G. Desarmenien systematically analyzed electrical activity of magnocellular neurons (although without distinction between OT and AVP) in the SON of rats during early postnatal life. Three main features arise from these studies: the importance of the second postnatal week (PW2), the role of autocontrol by OT, presumably released from soma and dendrites, and the determinant role of the development of electrophysiological features on the morphological maturation of the neurons. At birth and during PW1, the membrane potential is unstable and cells fire small and large erratic action potentials. During PW2, the membrane potential stabilizes to a more hyperpolarized value, the spontaneous activity becomes organized and the action potential progressively increases in size and decreases in duration, leading to a decrease in action potential-evoked calcium entry (Widmer et al., 1997; Chevaleyre et al., 2000). At the end of PW2 and during PW3, a switch in regulation of intracellular free calcium from extrusion to sequestration into the reticulum also occurs (Lee et al., 2007a). These two modifications of calcium entry and regulation may have important consequences on peptide secretion. The PW2 is also the period during which the chloride equilibrium potential becomes hyperpolarized, GABA becomes inhibitory and glutamatergic activity appears (Chevaleyre et al., 2001), together with an increase in NMDA receptor expression (Hussy et al., 1997). Most interestingly, this period of action potential and synaptic activity maturation is concomitant with the appearance of a major feature of SON neurons: autocontrol (Figure 3).

OTR has been shown to play an important role in autoregulation of magnocellular neuronal activity (Richard et al., 1997) and it is well established that OTRs are expressed in adult PVN (van Leeuwen et al., 1985; Freund-Mercier et al., 1987; Tribollet et al., 1988; Yoshimura et al., 1993; Adan et al., 1995). During ontogenesis, cells expressing OTR mRNA have been observed in SON and PVN starting from P1 (Yoshimura et al., 1996), a finding consistent with the faint and diffuse expression of OTR present throughout the hypothalamus at this stage (Tribollet et al., 1989). An increase in OTR mRNA expression is detected from P7 and is then maintained fairly stably to adulthood (Yoshimura et al., 1996).

Consistently with changes in OTR expression during PW2, OT and its related analog are most efficient in increasing electrical activity on one-third of SON neurons (supposed to be OT-ergic neurons since they were insensitive to AVP) and somatodendritic release of native OT (Chevaleyre et al., 2000).

The somatodendritic release of OT not only activates action potential firing, it is also determinant for the maturation of glutamatergic synaptic activity and of the neuronal morphology. Indeed at birth, supraoptic neurons display an oblong soma from which 2-3 dendrites with few proximal branches arise (Chevaleyre et al., 2001). However, during PW2, the interplay between incoming glutamatergic inputs and autocontrol induces an intense sprouting of dendritic branches (Chevaleyre et al., 2002), as if neurons were exploring the environment to establish new connections. This sprouting is transient and the neurons acquire their mature morphology (Randle et al., 1986) by the end of PW2. Although partial and concerning only SON OT neurons, these data point to a determinant role of autocontrol of OT neurons during PW2 in rats. This information should be taken into account in our understanding of how OT treatments during infancy can have lifelong consequences on OT-related social diseases (see the chapter below).

## Establishment of OT circuitry

Since neuroendocrinology was established as a new discipline, it has been clearly demonstrated that magnocellular (both OT and AVP) neurons of adult vertebrate species, including mammals, are primarily projecting to the posterior pituitary lobe to release these hormones into the systemic blood stream (Knobloch and Grinevich, 2014 and references therein). However, embryogenesis of pituitary OT projections remains unexplored. In fact, only two studies demonstrate such projection in rats without discrimination between OT and AVP components. The first study by the Ann-Judith Silverman group (Silverman et al., 1980) showed the existence of neurophysin-positive (i.e. without discrimination between OT and AVP) fibers in the posterior pituitary. Another study by the Michael Ugrumov and André Calas groups (Makarenko et al., 2000, 2002), employing DiI-based retrograde tracing in fixed brains, showed certain dynamics of these projections: first connections between the main part of the SON and pituitary are established earlier (detected at E15—earliest time point of the experiment), while the PVN and retrochiasmatic parts of the SON project to the pituitary later—at E17 (Makarenko et al., 2000). Intriguingly, the AN, composed mostly of OT neurons, projects to the pituitary only after birth (Makarenko et al., 2002).

Remarkable work on zebra fish larvae showed that OT is essential for the formation of an effective neurovascular interface (i.e., contacts of axonal OT terminals with fenestrated capillaries in the posterior pituitary; Gutnick et al., 2011). Unfortunately, such observation has not been extended to mice lacking OT or OTR, in which the analysis of the pituitary structure has not been reported.

Although central projections of OT neurons in adult rodents (mice, rats, and voles) have been significantly explored during last years (Ross et al., 2009; Knobloch et al., 2012; Dölen et al., 2013), the literature lacks reports on embryogenesis and early postnatal development of OT projections. This concerns both ascending projections of magnocellular OT neurons to the forebrain and descending projections of parvocellular OT neurons terminating in the brain stem and spinal cord.

The major inputs carrying visceral sensory information to the SON and PVN are relayed by catecholaminergic and non-catecholaminergic (glucagon-like peptide 1) neurons whose soma are located in the nucleus of the solitary tract and ventrolateral medulla. Both direct and indirect arguments indicate that these afferents are not functional at birth and develop during the first postnatal week (see review by Rinaman, 2007). Similarly, although no anatomical studies on the dynamics of innervation of OT neurons by glutamate and GABA have been performed, electrophysiological data summarized above suggest long-term postnatal maturation of synaptic inputs.

## The developmental trajectories of OT receptor expression

The developmental trajectories of OT receptor (OTR) expression in the nervous system have been investigated in mice, rats, voles and humans by different experimental approaches such as autoradiography, *in situ* hybridization and transcriptomic analysis, which provide complementary information. While autoradiography maps high affinity OTR at subcellular sites, which grossly correspond to receptor site(s) of activity on neuronal cell bodies and processes, *in situ* hybridization maps the neuronal bodies in which OTR mRNA accumulates. Transcriptomic analysis provides clues on brain regions actively synthesizing OTR mRNA without giving details on the cell types and subpopulations involved (see Table 2 for the available literature).

**Table 2:** **Studies of OTR trajectories in development**.

**Mice**	**Rats**	**Voles**	**Human**
Method: autoradiography (^125^I-OTA)	Method: autoradiography (^125^I-OTA)	Method: autoradiography (^125^I-OTA)	Method: transcriptomic analysis
Age: E18.5, P0, PN7, PN14, PN21, PN35, PN60	Age: PN1, PN5, PN10, PN14, PN18, PN24, PN60	Age: PN1, 1 week, 2 weeks, 3 weeks, 3 months	Age: 15 different periods of life from early embryonic (10-13PCW) to late adulthood (60Y+) Subject: Cerebellar cortex, mediodorsal nucleus of the thalamus, striatum, amygdala, hippocampus and 11 areas of neocortex (Kang et al., 2011)
Subject: all embryos and brain (Hammock and Levitt, 2013)	Subject: brain (Shapiro and Insel, 1989)	Subject: lateral septum (Wang and Young, 1997)	
Method: transcriptomic analysis	Method: autoradiography (^125^I-OTA)	Method: autoradiography (^125^I-OTA)	
Age: PN4, PN6, PN8, PN10, PN14, P180	Age: E12, E14, E16, E18, E20, PN1, PN3, PN5, PN10, PN13, PN16, PN19, PN22, PN25, PN30, PN35, PN40, PN45, PN60, PN90	Age: PN6, PN9, PN12, PN15, PN18, PN21, PN60	
Subject: primary somatosensory cortex (Fertuzinhos et al., 2014; http://hbatlas.org/mouseNCXtranscriptome/)	Subject: brain and spinal cord (Tribollet et al., 1989)	Subject: forebrain (Prounis and Ophir, 2013)	
	Method: autoradiography (^125^I-OTA) Age: E12, E14, E16, E18, E20, PN1, PN3, PN5, PN10, PN13, PN16, PN19, PN22, PN25, PN30, PN35, PN40, PN45, PN60, PN90		
	Subject: forebrain (Lukas et al., 2010)		
	Method: autoradiography (^3^H-OT)		
	Age: E20, PN1, PN5, PN15		
	Subject: brain (Snijdewint et al., 1989)		
	Method: *In situ* hybridization		
	Age: E9, E13, E14, E15, E17, E20, PN1, PN3, PN7, PN13, PN14, PN22, PN50 Subject: brain and spinal cord (Yoshimura et al., 1996)		

**Figure 4:**
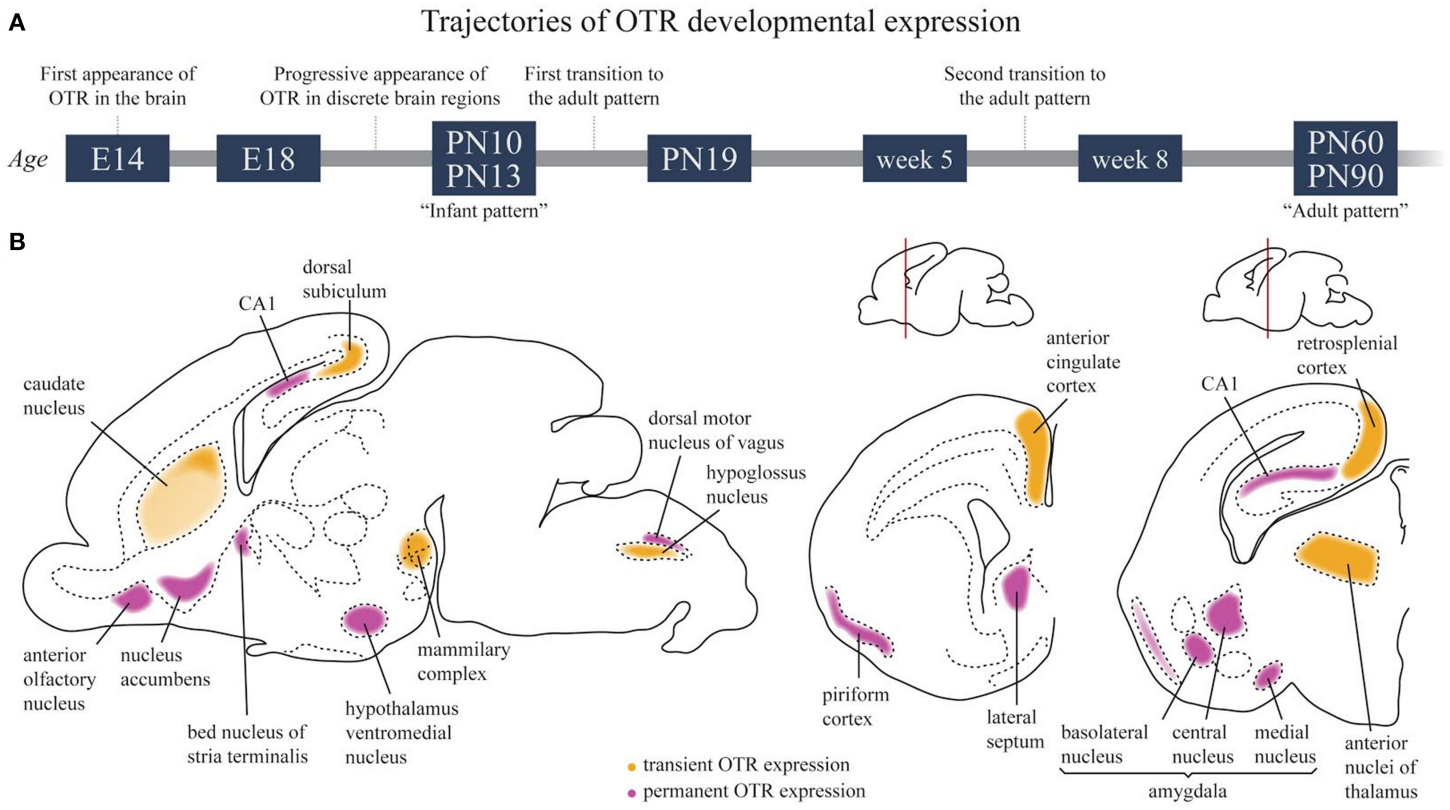
**Developmental trajectories of OTR in the rat brain**. **(A)** Schematic time course of OTR expression in the developing brain. **(B)** OTR expression in the infant brain around P10–P13. Regions in which a transient OTR expression is observed are colored in yellow; regions in which OTR expression is maintained to adult life are colored in magenta.

Among mammalian species, the rat has been by far the most extensively investigated. In this animal, the three aforementioned techniques resulted in highly comparable and consistent results, which made it possible to trace a developmental trajectory of OTR ontogenesis. This has been schematically summarized in Table 3 and Figure 4 (Shapiro and Insel, 1989; Snijdewint et al., 1989; Tribollet et al., 1989; Yoshimura et al., 1996; Lukas et al., 2010; Workman et al., 2013). Throughout the embryonic development and the first post-natal days, OTR progressively appears in several brain regions, reaching a well-defined “infant” pattern of distribution around PN10. After PN13, an abrupt decline of OTR density is observed in several areas, accompanied by expression in novel brain regions; this phase has been referred to as the first transition to the adult pattern and is basically completed at PN18. Around and after weaning, a second transition occurs, characterized by a novel reshaping of OTR expression, which slowly disappears from some areas and increases in others. Finally, the adult pattern of OTR expression is achieved at P60-90. Brain structures expressing OTR in different periods of prenatal and early postnatal life in rats (according to Shapiro and Insel, 1989; Tribollet et al., 1989; Lukas et al., 2010) are reported in Table 3.

**Table 3:** **Brain structures expressing OTR in different periods of prenatal and early postnatal life in rats**.

**Expression pattern**	**Structure**
Appearance in prenatal and/or early postnatal life and permanence to adult life	Dorsal nucleus of the vagus nerve
	Anterior olfactory nucleus
	Amygdaloid complex
	Nucleus accumbens°
	Dorsal peduncular cortex
	Lateral septum
	CA1 subfield of the hippocampus
	Ventral tegmental area
	Bed nucleus of the stria terminalis^*^
	Hypothalamic ventromedial nucleus^*^
	Ventral subiculum
Transient expression in prenatal and/or early postnatal life	Parietal cortex
	Cingulate cortex
	Retrosplenial cerebral cortex
	Mammillary complex
	Dorsal subiculum
	Caudate putamen
	Anterior and paraventricular thalamic nuclei
	Reticular nucleus
	Substantia gelatinosa of the V nerve
	Nucleus of the hypoglossus
Expression in late postnatal life	Olfactory tuberculum (Calleja islands)
	Ventral pallidum

° *In the adult, binding in the accumbens has been reported to almost completely disappear (Tribollet et al., 1989) or to be greatly reduced as compared to its pick at PN20 (Shapiro and Insel, 1989)*.

* *The appearance of OTR in the hypothalamic ventromedial nucleus has been reported to appear at PN1 (Tribollet et al., 1989) or to emerge only in the adult brain (Shapiro and Insel, 1989)*.

OTR appears as early as E14 pcd is the posterior portion of the neuronal tube that will become the vagal motor nucleus. Even though the mature OT is not detected until much later in development (see above), immature C-terminal extended forms of OT have been visualized much earlier (E16 pcd, see above). It is thus tempting to speculate that some immature OT forms may have an unrecognized role during early development in the vagal subregion. Mechanistically, the delivery of immature OT to the vagal nucleus can be achieved either via the neuropeptide diffusion through the brain tissue after somatodendritic release or via the transventricular pathway (Knobloch and Grinevich, 2014).

Strongly labeled areas of the “infant pattern” around PN10, schematically reported in Figure 4B, include the anterior olfactory nucleus, caudate putamen, accumbens, cingulate cortex, bed nucleus of the stria terminalis, some septal, thalamic and amygdaloid nuclei and the dorsal peduncular cortex. A faint but specific signal is observed at this stage in the hypothalamic ventral medial nucleus, a region that will become strongly labeled in the adult brain, while binding in the lateral septum and bed nucleus of the stria terminalis will undergo an intra-regional reshaping during post-natal development. Of relevance, between PN10 and PN25, is the disappearance of binding in the dorsal subiculum, accompanied by the concomitant appearance of increasingly strong binding in the ventral subiculum. During the same period, binding drops in the thalamus, cingulate cortex and CA1 but increases sharply in the accumbens, where it peaks at PN20 to subsequently decline during the second phase of transition to the adult brain. Changes in selected brain regions between week 5 and week 8 have been reported, such as an increase in OTR binding in the ventromedial hypothalamus and a decrease in the lateral septum (Lukas et al., 2010). However, a detailed, comprehensive study of OTR expression trajectories around weaning in males and females is, at present, missing.

Unfortunately, the studies available in literature for mice, voles and humans do not allow for similar comprehensive compilation of OTR expression trajectories during development. Nevertheless, a recent autoradiography study in mice revealed that region-specific trajectories of OTR expression are present in this species as well (Hammock and Levitt, 2013). A qualitative inspection of OTR binding from P0 to PN60 outlined a progressive strong increase of OTR from P0 to PN14, followed by region-specific up and down regulation of receptor expression. Different temporal expression profiles were reported in the three areas of the brain in which the OTR binding profile was quantified (hippocampus, lateral septum, and neocortex) with no significant differences between sexes. A very high degree of individual variability in OTR expression during ontogenesis was also reported in prairie voles, hampering the detailed delineation of developmental trajectories in this species; however, as observed in mice and rats, a higher density of OTR was observed in some forebrain regions (the septo-hippocampal nucleus and the hippocampus) between PN6 and PN21 as compared to PN1 and PN60 (Prounis and Ophir, 2013). Similarly, in the lateral septum, OTR density increased during the post-natal age, reaching the adult level at weaning; in particular, the binding increased more rapidly in mountain than in prairie voles, resulting in species differences at weaning and adulthood (Wang and Young, 1997). Finally, it is worth noting that in the different species, the overall pattern of the OTR expression during development does not match completely. The strong transient expression of OTR in the neocortex reported in mice was suggested to account for a species-specific role played by OTR during brain development (Hammock and Levitt, 2013). However, it should be mentioned that a transient OTR expression in the parietal cortex has also been reported in the rat brain (Snijdewint et al., 1989).

As in humans, the transcriptomic analysis of OTR reported a progressive increase in OTR mRNA during embryonic life in five out of six brain areas analyzed (Kang et al., 2011 and http://hbatlas.org/). Remarkably, the receptor level appeared to reach a maximum already before birth and to remain quite stable thereafter, at least in the first 5 years of life, although with some wide individual variations.

An important consideration when comparing developmental trajectories in translational medicine approaches is that the neurodevelopmental stage of the human brain at birth corresponds to rat and mouse brain at PN10 (Workman et al., 2013 and related web tool at http://www.translatingtime.net/home). Consistently, the human infant pattern of OTR expression is achieved before parturition while, in the rat, the infant pattern of OTR expression is achieved around PN10, and, in mice, between PN7 and PN14. To extrapolate the effects of pharmacological and environmental manipulation of the OT/OTR system in the human newborn brain from experiments performed in mice and rats, treatments should thus be performed in rodents around PN10, when a comparable maturation of the brain and of the OTR system has been reached in these species.

Several environmental factors have been reported to affect OTR expression in embryogenesis and early postnatal life, among which a predominant role is played by social and sensorial experiences, as more extensively discussed in the next paragraph. Furthermore, exposure to drugs and toxic agents can also modulate the OT/OTR system, as outlined by the interesting finding that nicotine and ethanol administration to pregnant rats increases OTR binding in the nucleus accumbens and in the CA3 region of the hippocampus of male offspring (Williams et al., 2009). Recently, an up-regulation of OTR in the nucleus accumbens, medial anterior olfactory nucleus and central and medial nuclei of amygdala has been reported in μ-opioid receptor knock out mice (Gigliucii et al., 2014), which suggests a close link between OTR expression and the opioid reward system.

## OT and social experience in development

Effects of OT during embryogenesis and early postnatal ontogenesis on social life are extensively summarized in recent reviews (Carter, 2014; Hammock, 2014), but the reverse—i.e., the effects of social stimuli on maturation of OT system—are less explored. It has been reported that early social experience tremendously affects physiology of the OT system (Bales and Perkeybile, 2012; Hammock, 2014). Recently it has been demonstrated that early sensory experience (in the newborn) regulates development of sensory cortices via OT-signaling (Zheng et al., 2014).

This oxytocin early regulation may have long-term consequences in adults. It has been known for many years that exogenous OT in neonates can revert the long-term behavioral effects of prenatal stress (Lee et al., 2007b) and has consequences on other endocrine systems (i.e., the estrogen receptor, Pournajafi-Nazarloo et al., 2007) as well as on blood pressure (Holst et al., 2002) in adults. The effects of OT, or of the environmental and familial circumstances resulting in increased production of mature neonatal OT, on adult social behavior mainly remain to be investigated. With respect to OTR expression, increased levels have been observed in offspring after communal rearing (Curley et al., 2012), increased maternal licking/grooming (Champagne et al., 2001) and social enrichment (Champagne and Meaney, 2007). On the contrary, late weaning has been reported to reduce OTR density in selected, socially relevant, brain regions (Curley et al., 2009). Furthermore, maternal separation has been found to induce a complex modulation of OTR expression with region-specific up and down regulation of OTR (Lukas et al., 2010). It is important to note, at the end of this section, that exploration of early life experience on OT/OTR system in animals provides a scientific basis for child care and new therapeutical approaches to ameliorating social alterations occurring in adult patients afflicted with Autism Spectrum Disorders or the PWS (see below).

## OT and developmental neurological disorders

Many reviews are exhaustive on the involvement of OT in shaping and regulating the social brain (Meyer-Lindenberg et al., 2011; Striepens et al., 2011; McCall and Singer, 2012) or in learning and memory (Chini et al., 2014). The strongest line of demonstration of the OT system in social behavior, feeding behavior and maternal care is via the study of knockout mice in which either the *OT* or the *OTR* genes are inactivated (Table 4). Mice constitutively lacking OT (*Oxt*^−/−^) are unable to release milk and have impaired social memory (Ferguson et al., 2000). Mice with constitutive ablation of the OTR gene (*Oxtr*^−/−^) display a behavior very similar to the *Oxt*^−/−^ mouse, mainly marked by social deficits (Takayanagi et al., 2005). In addition, although learning is normal in *Oxtr*-/- mice, reversal learning is strongly decreased, indicating impaired cognitive flexibility reminiscent of the ASD syndrome (Sala et al., 2011). Importantly, even a 50% loss of the OTRs also leads to an impairment of social behavior, suggesting that a fine tuning of the OT system is necessary to control behavior (Sala et al., 2013). Other mouse models, in which the knock-out of a specific gene induced a disruption in the OT system that has been linked to a pathological phenotype, reinforce the role of OT in neurodevelopmental disorders (Table 4). Recently, it has been shown that disruption of the neonatal surge of OT coming from the mother during delivery results in autistic-like features in the adults (Tyzio et al., 2014). All these data suggest that an early postnatal injury or dysfunction of the OT system has consequences in infant and adult behaviors. Unfortunately, the contribution of early life experience to OT signaling, in a pathological context or following a trauma, has not been extensively studied in the brain of highly social mammalian species such as voles or monkeys. A limited number of reports (Bales et al., 2007, 2011; Ahern and Young, 2009) demonstrated that social deprivation and enrichment paradigms induce changes in OT synthesis and OTR binding in voles (Bales et al., 2007, 2011; Ahern and Young, 2009). While it is not yet clear how these changes occur, the most likely scenario is the environmental influence on the gene expression through epigenetic mechanisms.

**Table 4:** **OT and neurodevelopmental disorders**.

**Alteration**	**Mouse models**	**Human (early neurodevelopmental disorders)**
Feeding problems:	*Magel2* KO (Schaller et al., 2010)	Prader-Willi syndrome (McAllister et al., 2011; Miller et al., 2011)
Early impairment of feeding (suckling activity) Obesity	*Oxt*-KO (Camerino, 2009)	Autistic patients with *MAGEL2* mutations (Schaaf et al., 2013)
	*Oxt*-KO (Takayanagi et al., 2008)	
	*Sim1*-KO (heterozygotes) (Kublaoui et al., 2008)	
Social and emotional defects ASD	*Oxtr*-KO (Takayanagi et al., 2005; Sala et al., 2011, 2013)	ASD (Li et al., 2012; Aoki et al., 2014; LoParo and Waldman, 2014)
	*Oxt*-KO (Ferguson et al., 2000)	Prader-Willi syndrome (Swaab et al., 1995; Dykens et al., 2011; Tauber et al., 2011)
	*CD38*-KO (Jin et al., 2007)	Williams syndrome (Dai et al., 2012; Jarvinen and Bellugi, 2013)
	BTBR T+ tf/J (Silverman et al., 2010)	Autistic patients with *MAGEL2* mutations (Schaaf et al., 2013)
	*Magel2* KO (FM, submitted)	
	*FraX* KO (Mineur et al., 2006; Pietropaolo et al., 2011; Francis et al., 2014)	
	*MageD1*-KO (Dombret et al., 2012)	
	Haploinsufficient *reeler* (Liu et al., 2005)	
Maternal care and/or social development of the progeny	*Oxt*-KO (Winslow et al., 2000)	*OTR* risk allele
	*CD38*-KO (Liu et al., 2008)	*CD38* risk allele (Feldman et al., 2012; Rilling and Young, 2014)
	*Oxtr*-KO (Higashida et al., 2010; Rilling and Young, 2014)	
	*Peg3*-KO (Champagne et al., 2009)	
Neurodegenerative diseases	*CD157(BST1)* KO Lopatina et al., 2014	No clear data

In humans, autistic spectrum disorder (ASD) is a broadly-defined disorder that mainly affects behavior and cognition. Social interaction impairments are the most characteristic deficits in ASD. Interestingly, several lines of evidence suggest a role of OT in the etiology of ASD (Harony and Wagner, 2010; LoParo and Waldman, 2014) and OT's therapeutic effects have been observed in social communication (Aoki et al., 2014), however, they are still debated (Guastella et al., 2014). Furthermore, it is also acknowledged that an alteration in the OT-system might be involved in neurodevelopmental disorders marked by social cue deficits such as Fragile X Syndrome, Williams Syndrome and PWS (see Table 3 and Francis et al., 2014).

**Figure 5:**
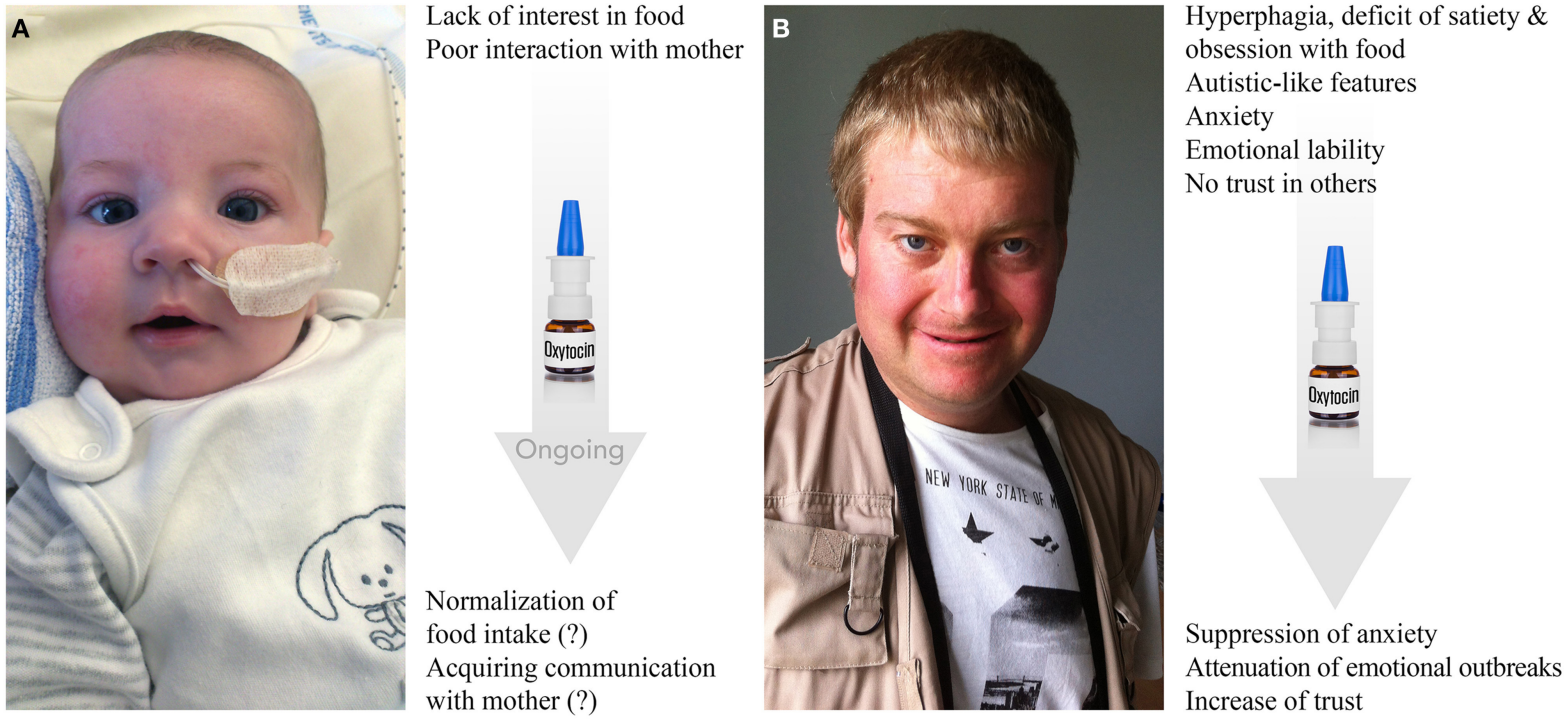
**Prader-Willi syndrome: clinical features and effects of OT**. **(A)** Three-month-old baby born at term has symptoms of hypotonia, deficit of suckling and poor interest in food. Nasogastric tube, which is shown in the photo, is used in almost all PWS neonates to prevent failure to thrive for a mean of 1 month. Baby also exhibits facial dysmorphism, such as almond-shaped eyes, thin upper lip, down-turned corner of the mouth, narrow bi-frontal diameter and slight facial assymetria. Behaviorally, the baby shows low interest in mother and poor social skills in general. Ongoing treatment with OT shows positive trends in both stimulation of food intake and social bonding with the mother (further confirmation is needed). **(B)** Twenty-three-year-old male patient has a record of hyperphagia, deficit of satiety, obsession with food, dysmorphic features (scoliosis, kyphosis), autistic-like features (stereotype and repetitive movements, deficit of social skills and poor trust in others), anxiety, emotional liability (including emotional outbreaks), creation of stories filled with fantasies (termed as “fabulation”) and compulsive skin picking. The patient is under psychotropic treatments. First applications of OT seem to result in increase of trust, decrease in anxiety and attenuation of emotional outbreaks (further confirmation is needed). Photos are reproduced here with permission of Prof. Maithé Tauber.

PWS is one of the best reported examples of a neurodevelopmental disease characterized mainly as an eating disorder with behavioral and social disturbances (Figure 5; Butler et al., 2011; Dykens et al., 2011; McAllister et al., 2011; Cassidy et al., 2012; Jauregi et al., 2013). PWS is a rare genetic disease with an estimated prevalence worldwide of 1 in 10,000–30,000 individuals (Cassidy et al., 2012). Patients with PWS exhibit a complex and progressive phenotype. Their eating behavior is mainly characterized by two opposite stages (Butler et al., 2010; Miller et al., 2011). During phase I of the syndrome, apparent at birth, the suckling activity is weak or absent and babies show little interest in feeding during the first few months of their lives. After 2 years, it is characterized by a true hyperphagia with obesity. In fact, PWS children initially display anorexia as neonates and then switch to hyperphagia with obesity (Tauber et al., 2014).

In parallel with the eating problem, PWS patients have mild to moderate intellectual disability and behavioral alterations (Ho and Dimitropoulos, 2010; Chevalere et al., 2013), including emotional outbreaks (temper tantrums) and compulsive traits (Dimitropoulos et al., 2006). Repetitive and ritualistic behaviors and difficulty with routine changes (Holland et al., 2003; Greaves et al., 2006; Dykens et al., 2011), similar to those found in autistic spectrum conditions, have also been described. Indeed all PW patients share some features of ASD (Koenig et al., 2004). Some patients, who are diagnosed as autists, share some features observed in PWS and are defined as PWS-like patients (Schaaf et al., 2013).

Nevertheless, only a few patients with PWS are diagnosed as autists. Patients with PWS present greater overall behavior disturbance than age-matched mentally retarded patients, but score comparably to patients with psychiatric disorders. Indeed, they frequently display anxiety traits and anxious mood comparable to those of patients presenting anxiety disorder or schizophrenia. They also show pronounced emotional liability and a striking inability to control their emotions, which results in frequent emotional outbreaks (temper tantrums), which can occur due to an impaired capacity to understand the motivations of others in the social environment, possibly indicating deficits in “theory of mind” (the ability to attribute mental states to others) and empathy (the ability to infer emotional experiences) (Lo et al., 2013). Importantly, Tauber et al. (2011) reported the first clinical trial on OT that showed that a single intranasal administration of OT rescue some behavioral features, such as increased trust and decreased signs of depression as well as emotional outbreaks, in patients with PWS (Figure 5). However, a double-blind randomized cross-over trial of OT nasal spray performed in 22 PWS patients (12–29 years old) did not find statistically significant effects using 18–40 IU of intranasal OT twice daily for 8 weeks (Einfeld et al., 2014). The authors reported an increased number of temper tantrums in the patients receiving the high dose of OT and no effect was observed in patients treated with low doses. The discrepancies between these two studies may be explained by the dose used, the short wash-out period (15 days), the duration of treatment and the sex ratio (most patients were male in the report of Einfeld and colleagues compared to the report of Tauber's team).

Much of the phenotype of PWS is consistent with a hypothalamic defect (Swaab et al., 1995; Swaab, 1997a,b) characterized by a reduction of OT expressing neurons in the PVN, mostly represented by parvocellular OT neurons (Swaab et al., 1995; Swaab, 1997a,b). These cells project to the brainstem nuclei, including the nucleus of the solitary tract, where OT acts as a powerful anorexic peptide (Atasoy et al., 2012). Normal OT plasma levels have been detected in 17 PWS adult patients (Höybye et al., 2003), but elevated cerebrospinal fluid (CSF) OT levels have been reported in five PWS patients (Martin et al., 1998). This high level of OT in the CSF most likely relies on OT released from magnocellular neurons, contacting both the vasculature of the posterior pituitary and the lumen of the third ventricle (Knobloch and Grinevich, 2014). However, such discrepancy between OT released into the blood and CSF needs further clarification, especially with respect to methodological limitations for measuring OT in biological samples (McCullough et al., 2013).

Genetically, PWS results from the lack of expression of several contiguous imprinted genes located in the 15q11–q13 region. These genes are regulated by genomic imprinting: a mechanism leading to the transcriptional expression of the paternal alleles of these genes only, the maternal alleles being silent (i.e., not expressed). Recently, pathogenic mutations of *MAGEL2* alone have been reported in four patients (Schaaf et al., 2013), causing a classical PWS in one patient and PWS-like phenotypes in the other three. All of the cases described were also diagnosed with early feeding problems and ASD, without severe obesity. These results underline *MAGEL2's* contributing role in cognitive and behavioral alterations in PWS and early feeding in general.

Francoise Muscatelli's group created a mouse model deficient for *Magel2*. These mice showed an altered onset of suckling activity and subsequent impaired feeding leading to 50% of neonatal lethality, affecting both males and females (Schaller et al., 2010). Impressively, there is an obvious alteration of production of mature OT in the PVN of *Magel2*-deficient pups, while a single administration of OT, in a restricted time window after birth, allows resetting of the feeding behavior and consequently rescues the life of all pups (Schaller et al., 2010, p. 24). Furthermore, inactivation of *Magel2* induces in adult males (but not in females) a deficit in social recognition and social interaction as well as a reduced learning ability with an alteration of social and spatial memory. A daily administration of oxytocin (OT) in the first week of life is sufficient to restore suckling activity at birth and to restore a normal social behavior and learning abilities in adult mutant males (Meziane et al., in press).

Altogether, these results suggest that an alteration of the OT system around birth has early and long term consequences on feeding and social behaviors and on cognition. Importantly, an OT treatment of Magel2-deficient pups in the first post-natal week partially restores a normal anatomy of the OT system and prevents deficits in social behavior and learning in adults. This concept opens the door to a powerful pharmacological therapy in early infancy for the PWS and might be considered for other pathologies such as autism spectrum disorders. Importantly, a clinical trial on OT treatment of PWS infants has been initiated by Maithé Tauber and her team.

## OT pathways in perinatal ontogenesis: physiology, pathology, and treatment

**Figure 6:**
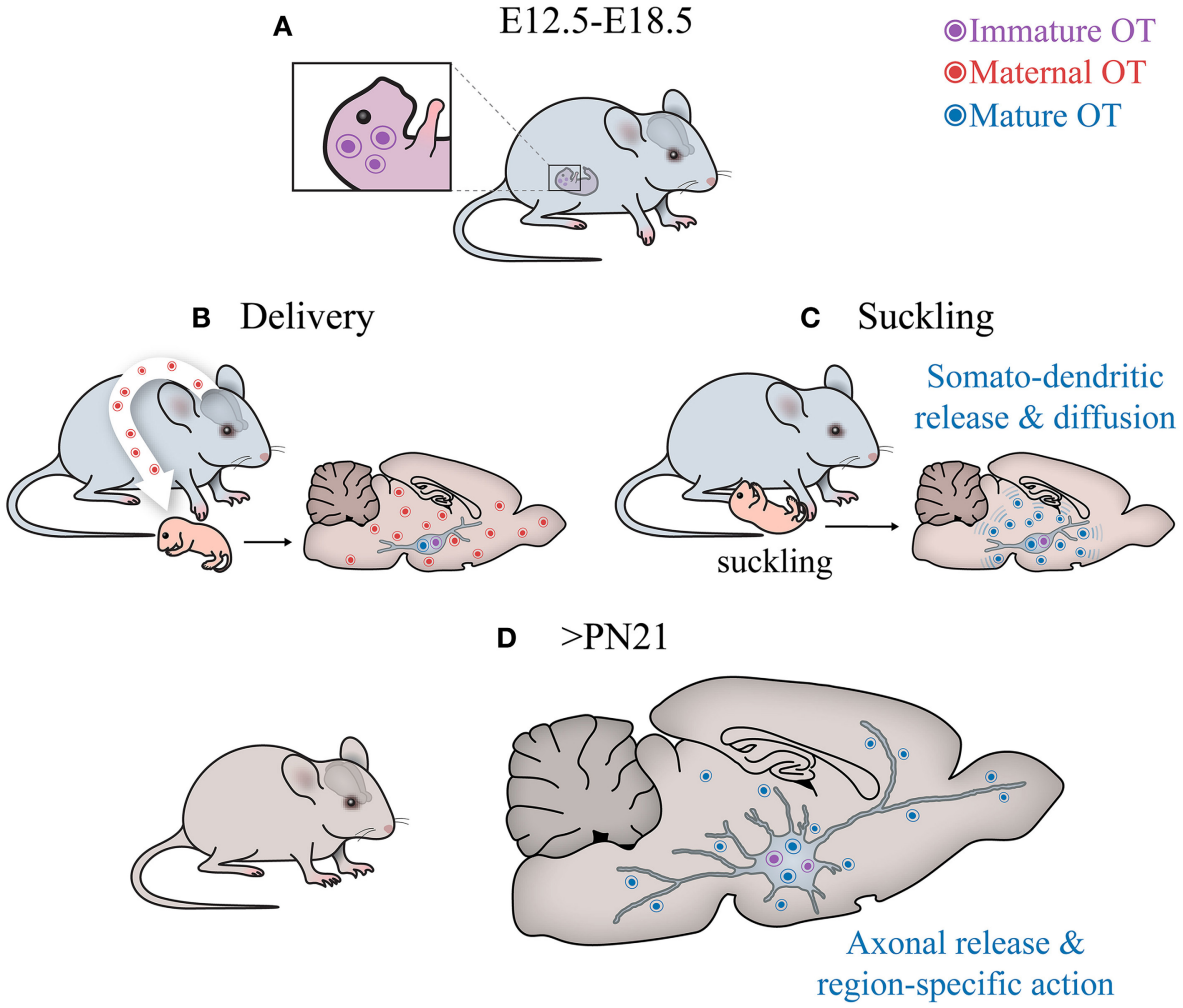
**Central OT pathways in development**. **(A)** The hypothalamus of embryos produces immature OT. **(B)** After birth, the cells start to generate mature (amidated) OT. Although it is unclear why this switch occurs, the fact that it happens after parturition suggests that perinatal maternal OT (of extremely high concentrations!), passing via the placental barrier (Malek et al., 1996) to the hypothalamus of newborns, may initiate the activation respective enzyme (see Figure 2) and, hence, appearance of the mature form of OT. **(C)** After birth, the suckling activity of pups transmitted to the activation of sensory pathways converging on OT neurons, may stimulate somatodendritic OT release. Such mode of release leads to “ubiquitous filling” of the brain with OT. **(D)** Later on, starting from the fourth week, OT neurons establish axonal projections to various brain areas to release OT into selective targets to modulate specific behaviors, especially social behaviors (Knobloch and Grinevich, 2014).

While the picture of OT pathways development is far from complete, at least three conclusions can be made (see Figure 6):

1. OT neurons start to produce a mature form of OT in rodents much later than other neurons express AVP and hypophyseotrophic neuropeptides (Markakis and Swanson, 1997; Markakis, 2002). In fact, in mice, the mature form of OT appears on the day of delivery, suggesting that maternal OT can promote synthesis of mature OT in newborns.
2. OT neurons (magnocellular) at birth have immature morphological (“spindle-like morphology”) and physiological properties (including intrinsic electrophysiological properties and synaptic inputs). Furthermore, many OT neurons project axons to the posterior pituitary only after birth (Makarenko et al., 2002) and no collaterals of OT axons have been reported in extrahypothalamic brain regions of neonates in contrast to those in adult rodents (Knobloch and Grinevich, 2014).
3. OT receptors are widely expressed in the brain well before the synthesis of mature OT, suggesting their receptivity either for immature forms of OT, maternal OT, maternal/neonatal AVP or uncharacterized peptides. During maturation of the system (i.e., postweaning period > P60), the expression of OTR is restricted to several brain regions, most of which are innervated by OT axons at this age (Knobloch et al., 2012; Dölen et al., 2013).

The delayed maturation of the central OT system in rodents (and humans) may be caused or may correlate with the feature of these species, which are born relatively immature (termed “altricial” species). From a comparative point of view, it would be interesting to compare the dynamics of OT system maturation with the animals which are relatively mature and mobile from the moment of birth (termed “precocial” species, such as guinea pigs).

Since the distant/extrahypothalamic OT axons during neonatal period in studied (altricial) species are absent (at least not reported), but OT neurons were responsive to externally applied OT, it is most likely that neonate rodents operate by endogenous OT released from somato-dendritic compartments of OT cells. While PVN OT cells are closely located to the ependymal layer of the third ventricle, OT may diffuse also to the cerebrospinal fluid, resembling the evolutionarily old “transventricular” pathway of OT action (Knobloch and Grinevich, 2014). Moreover, OT can reach the brain directly via brain capillaries, as the blood brain barrier is not formed at that age (Ugrumov, 2010 and references therein), allowing peripherally administered OT to efficiently reach the brain (Meziane et al., in press).

In line with the “diffusion-like” mode of OT action, the report on newborn and young mice (P0–P14; Zheng et al., 2014) showed no axonal projections in the somatosensory cortex, while the effects of OT on electrical activity of cortical neurons as well as on sensory processing were very prominent (Zheng et al., 2014). Keeping this contradiction in mind, the authors speculated about “diffusible” OT reaching the cortex from the hypothalamus. Despite the lack of literature, it is tempting to speculate that magnocellular OT axons will grow to forebrain regions and parvocellular OT neuronal axons to brain stem/spinal cord only after weaning, to execute addressed OT release aimed to orchestrate autonomic and behavioral responses respectively (see above).

Animal models of social deficiency (such as OT and OTR knockout mice) and human cases of OTR gene duplication (Bittel et al., 2006) support the critical role of neonatal OT signaling in the development of an adequate social skill. The model of PWS—Magel2 knockout mice has pronounced OT deficiency with delayed expression of mature OT on the day of birth.

The discovery that peripheral OT administration to neonate Magel2 knockout mice rescues both the feeding problem in neonates and social impairments in adults, gives the reason to dissect alteration in sequences of OT signaling (i.e., in steps of OT expression in OT cells, OT transport via axons and their releasing capacity, expression of OTR etc.), which can be considered as target(s) for exogenous OT. Keeping in mind the potential OT sensitivity of OT neurons, and many other brain cells, as well as long-lasting OT effects, someone may hypothesize that exogenous OT stimulates transcriptional, electric and secretory activity of OT neurons, which start to release larger amounts of OT to fill in the brain. Additionally, upregulation of central OTR expression can facilitate OT signaling. However, the testing of these scenarios requires further comprehensive research.

### Conflict of interest statement

The authors declare that the research was conducted in the absence of any commercial or financial relationships that could be construed as a potential conflict of interest.

## Footnotes

^1^Parvocellular OT neurons belong to a relatively small population of PVN cells, which project to the brainstem and spinal cord (Sawchenko and Swanson, 1982) and control appetite, nociception and autonomic functions. The development of these particular neurons has not been studied.
